# Case Report: A classical PSGN case with unusually prominent serosal manifestations and complement patterns that mimicked systemic autoimmune disease—highlighting diagnostic pitfalls and biopsy decision-making

**DOI:** 10.3389/fped.2026.1759332

**Published:** 2026-03-09

**Authors:** Vinson James, Sona Santy, Teresa Saverimuttu, Vindhya Kamath, Julie Steinberg

**Affiliations:** 1 Department of Pediatrics, Good Samaritan University Hospital, West Islip, NY, United States; 2 New York Institute of Technology College of Osteopathic Medicine, Old Westbury, NY, United States

**Keywords:** diagnostic uncertainty, hypocomplementemia, pediatric nephritic syndrome, post-streptococcal glomerulonephritis, serosal involvement

## Abstract

**Introduction:**

Post-Streptococcal glomerulonephritis (PSGN) most commonly follows streptococcal infections and presents with classic features such as hematuria, proteinuria, hypertension, and transient renal dysfunction. While renal-limited disease is typical, extrarenal manifestations—particularly serosal involvement—are exceptionally rare in children.

**Case presentation:**

We report a rare case of an adolescent who presented with nephritic syndrome marked by hypertension, gross hematuria, proteinuria, and notably, concurrent pleural and pericardial effusions. Laboratory evaluation revealed low serum complement levels (C3 and C4), consistent with immune complex-mediated glomerulonephritis. Extensive infectious and autoimmune workups were unremarkable. The patient was managed conservatively with antihypertensives and diuretics, with complete resolution of symptoms and normalization of renal function and complement levels within four weeks. To our knowledge, serosal involvement (pleural and pericardial effusions) at initial presentation in pediatric PSGN remains extremely rare, with very few documented cases in the literature. This report contributes valuable clinical insight, emphasizing that PSGN can occasionally mimic systemic inflammatory or autoimmune conditions. Early identification and conservative management can prevent overtreatment and improve outcomes.

**Conclusion:**

This case underscores an unusual presentation of PSGN with serosal involvement—a manifestation reported only sporadically in literature. Recognition of such rare systemic features is crucial to avoid diagnostic delays or unnecessary immunosuppression. Supportive care alone led to favorable outcomes, reinforcing the self-limited nature of PSGN even in atypical presentations. It serves as a valuable reminder that atypical PSGN can present with multi-system inflammation, and a precise diagnostic approach integrating serology and clinical course is essential to avoid unnecessary intervention.

## Introduction

Post-infectious glomerulonephritis (PIGN) is a well-recognized cause of acute nephritic syndrome in children and adolescents, most commonly triggered by group A streptococcal infections. The classic presentation includes hematuria, proteinuria, hypertension, and transient impairment of renal function, often accompanied by hypocomplementemia—particularly low C3 levels. While renal manifestations dominate the clinical picture, systemic features such as serosal involvement (pleural or pericardial effusions) are exceedingly rare. Systemic features such as pleural or pericardial effusions are exceedingly rare in PSGN and may create diagnostic uncertainty, particularly when cardiopulmonary symptoms are prominent. We report a pediatric case of PSGN presenting with concurrent pleural and pericardial effusions, highlighting the challenges in distinguishing severe renal volume-related manifestations from systemic inflammatory disease. In such cases, overlapping signs of volume overload and inflammation may obscure the diagnosis, especially when cardiopulmonary symptoms are prominent. We report a rare and severe presentation of PIGN with concurrent pleural and pericardial effusions in a pediatric patient. This case highlights the diagnostic complexity and underscores the importance of maintaining a high index of suspicion for atypical manifestations of PIGN. The case highlights the diagnostic challenge when classic PSGN features coincide with findings suggestive of broader systemic inflammation.

## Case presentation

A 12-year-old Hispanic female with a past medical history of asthma presented to the emergency department (ED) with a four-day history of abdominal pain, chest tightness, and shortness of breath. Her medical history was notable for a congenital aberrant right subclavian artery, for which she underwent surgical reimplantation when she was two years old. She had been followed regularly by cardiology, with her most recent evaluation one year prior showing no abnormalities. The patient was allergic to penicillin, had no other surgical history, and had no significant family history. Prior to ED arrival, her mother administered ibuprofen (Motrin) and the patient used her albuterol inhaler without relief. She denied recent fever, cough, congestion, sore throat, sick contacts, or recent travel.

In the ED, the patient's blood pressure was markedly elevated at 159/111 mmHg, compared to normal readings recorded by her primary care provider one month earlier. Her respiratory rate was 22 breaths per minute, and other vital signs were within normal limits. On examination, she exhibited mild increased work of breathing but was able to speak in full sentences. Bilateral periorbital edema and facial swelling were noted. Lung auscultation revealed diminished beath sounds at the bases, no wheezing/ crackles, and +1 pitting edema was present in the lower extremities up to the level of the calves. She required supplemental oxygen for increased work of breathing and desaturation to eighty eight percent at one liter via nasal cannula. The remainder of her physical examination was unremarkable.

Initial evaluation included urinalysis, which was ordered to assess for urinary tract infection but instead revealed large amounts of red blood cells and protein (300 mg/dL). These findings were concerning for nephrolithiasis in this pre-menarchal patient, thus a non-contrast CT scan of the abdomen and pelvis was performed. The CT scan did not show any renal pathology, but revealed mild cardiomegaly with trace pericardial effusion, moderate bilateral pleural effusions, and mild pelvic free fluid. An echocardiogram showed no abnormalities.

**Figure 1:**
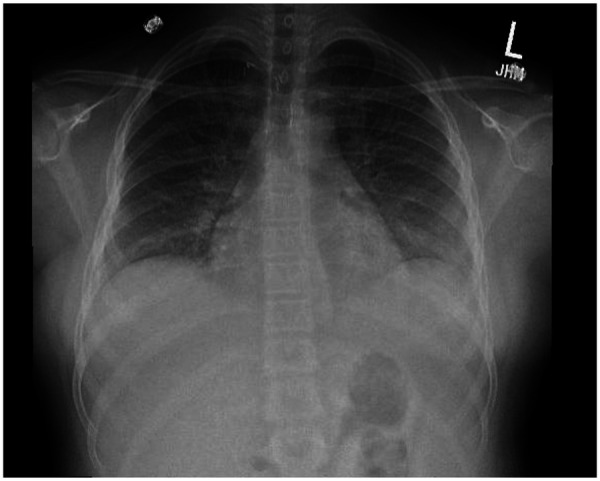
Chest xray PA view demonstrating bilateral pleural effusions and trace pericardial effusion at presentation.

**Figure 2:**
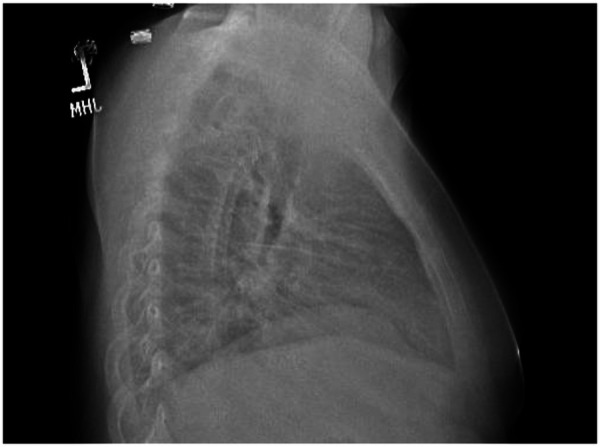
Chest xray lateral view demonstrating bilateral pleural effusions and trace pericardial effusion at presentation.

**Figure 3:**
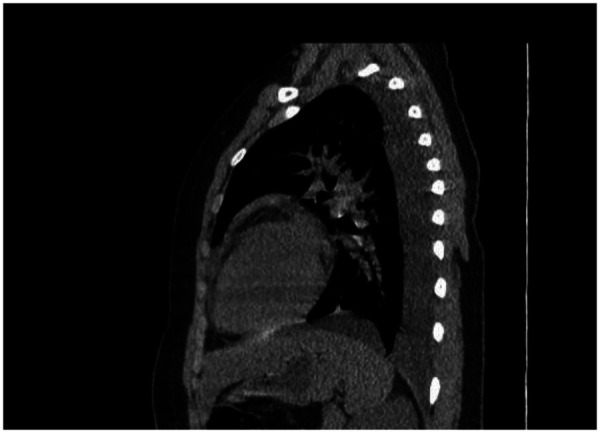
Non- contrast CT chest demonstrating bilateral pleural effusions and trace pericardial effusion at presentation.

Given the new-onset hypertension and proteinuria, laboratory evaluation was pursued for possible nephritic or nephrotic syndrome. The patient's complete blood count showed elevated white blood cell count with lymphocytosis. A respiratory viral panel was positive for SARS-CoV-2. Her comprehensive metabolic panel revealed hypoalbuminemia (albumin 2.9 g/dL), creatinine (0.57 mg/dL), peak urine protein/creatinine ratio of 4,422 mg/g and elevated alkaline phosphatase (168 U/L). Antistreptolysin O (ASO) titers were elevated at 1,547 IU/mL, while complement C3 and C4 levels were markedly decreased (24 mg/dL and 11 mg/dL, respectively). Although antinuclear antibodies were positive, dsDNA was negative, and she lacked other laboratory workup concerning for lupus nephritis. Based on the combination of elevated ASO titers, decreased complement levels, hypertension, and proteinuria, a diagnosis of poststreptococcal glomerulonephritis (PSGN) was made (Figures 1–3).

Management included initiation of 1 L/min supplemental oxygen by nasal cannula for desaturation to eighty eight percent and increased work of breathing with tachypnoea and nasal flaring. She was started on intravenous furosemide (Lasix) 20 mg (two doses), and amlodipine 2.5 mg daily as recommended by cardiology. Overnight, the patient's oxygen saturation dropped to 86%, necessitating an increase in nasal cannula flow to 3 L/min and administration of a third dose of furosemide. By hospital day two, the patient reported significant improvement in chest tightness and abdominal pain, was able to ambulate without dyspnea, was weaned to room air, and showed improvement in periorbital and lower extremity edema, although diminished breath sounds persisted. Due to persistent hypertension, amlodipine was increased to 7.5 mg daily. The patient was discharged on hospital day four with a prescription for amlodipine 7.5 mg daily and instructions to follow-up with nephrology within one week. Repeat labs before discharge demonstrated stable renal function and electrolyte balance, and urinalysis showed decreased proteinuria. Her labs showed an improving trend of proteinuria and hypoalbuminemia with urine protein/creatinine: 1,197 mg/g, and serum albumin: 3.2 g/dL. At nephrology follow-up at 3-week nephrology clinic visit, the patient's shortness of breath had completely resolved, and her blood pressure was only mildly elevated, allowing discontinuation of antihypertensive therapy. She had normalized labs, including albumin (3.9 g/dL), complements (C3: 153 mg/dL, C4: 22 mg/dL), and Urine protein to creatinine ratio had normalized (<125 mg/g). She was scheduled for continued outpatient follow-up.

## Discussion

Post-infectious glomerulonephritis (PIGN), particularly acute post-streptococcal glomerulonephritis (APSGN), remains a common cause of nephritic syndrome in children and generally follows a self-limited course (1). It typically arises 1–3 weeks after streptococcal pharyngitis or skin infection and is characterized by hematuria, edema, hypertension, and varying degrees of renal dysfunction (2). Most children recover fully with supportive care. This case highlights atypical clinical features that expanded the initial differential diagnosis despite serologic evidence of recent streptococcal infection.

### Atypical clinical features

While hematuria, edema, and hypertension are common in APSGN—with reported rates approaching 100%, 65%–90%, and 80%–90% respectively—our patient exhibited multiple rare features: nephrotic-range proteinuria, severe hypertension, Low complement C4 and dual serositis (pleural and pericardial effusions) (3–5). Nephrotic-range proteinuria occurs in only 5%–10% of cases, and although pleural effusion may result from fluid overload, pericardial involvement is highly unusual. Pleural effusions in nephritic states are frequently attributable to volume overload and hypoalbuminemia. Pericardial effusion, while less common, can also occur in settings of fluid overload, hypertension, and hypoalbuminemia. In this case, severe hypertension, fluid retention, and hypoalbuminemia likely contributed to the development of serosal effusions, although their extent at presentation contributed to concern for systemic inflammatory disease (6–8). This suggests that, in addition to hemodynamic factors, a component of immune-complex mediated serosal inflammation—reflected by the profound systemic hypocomplementemia—may have contributed to the effusions, making the presentation particularly dramatic.

### Complement pathways and diagnostic implications

Complement activation is central to the pathogenesis of acute post-streptococcal glomerulonephritis and typically involves the alternative pathway, resulting in depressed serum C3 levels with normal C4 in most cases. However, early or severe disease may transiently activate the classical pathway, leading to concurrent reductions in C3 and C4. In this patient, the presence of combined hypocomplementemia initially raised concern for autoimmune glomerulonephritides; however, the marked elevation in antistreptolysin O titers, absence of disease-specific autoantibodies, and spontaneous normalization of complement levels within four weeks supported a diagnosis of post-streptococcal glomerulonephritis rather than immune complex–mediated systemic disease.

### Inflammatory markers and prognosis

While APSGN typically has a favorable prognosis, studies indicate that some children may develop reduced glomerular filtration rate (GFR) during the acute phase (9). In a retrospective study of 75 pediatric APSGN patients, decreased GFR was significantly associated with hypoalbuminemia, elevated C-reactive protein (CRP), and particularly low C4 levels. These findings suggest that C4 hypocomplementemia and systemic inflammation may serve as markers of more severe renal involvement (10–12). Our patient's constellation of low C4, nephrotic-range proteinuria, and high inflammatory markers further supports this correlation and highlights the need for vigilant monitoring.

### Timeline of clinical recovery

**Figure 4:**
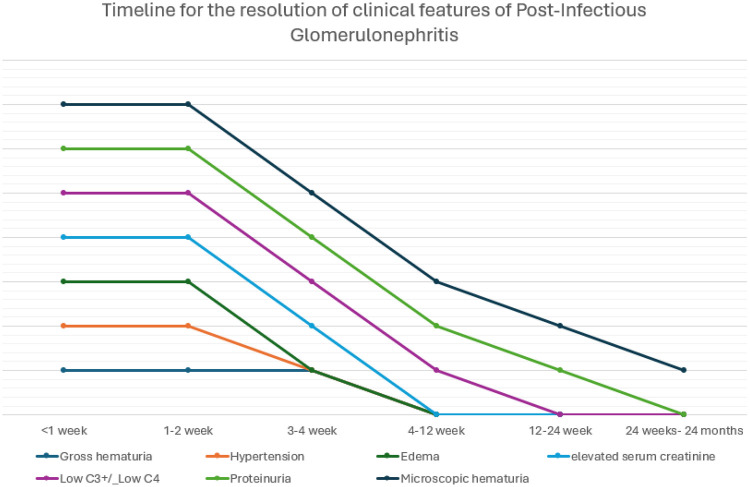
Timeline for the resolution of clinical features of post-infectious glomerulonephritis (PIGN).

Most clinical and laboratory abnormalities in APSGN resolve spontaneously. Edema and hypertension resolve within 1–2 weeks. Elevated creatinine and gross hematuria typically normalize within 3–4 weeks. C3 returns to baseline by 6–8 weeks. Persistent complement abnormalities beyond this window should prompt evaluation for other glomerulopathies. In this case, serial monitoring demonstrated recovery in line with expected APSGN trajectory, confirming the diagnosis despite its atypical features (Figure 4).

### Atypical PIGN

While most cases of PIGN resolve with infection clearance, a subset of patients develops persistent or progressive glomerular disease without clear evidence of a preceding infection. Recent studies have identified abnormalities in the alternative complement pathway, such as genetic mutations or C3 nephritic factors, as a common feature in these atypical presentations (12). This case illustrates an atypical presentation of PSGN with features that broadened the initial differential diagnosis (13, 14). This possibility should be considered in patients with persistent hypocomplementemia, prolonged renal dysfunction, or histopathologic features inconsistent with resolving PIGN.

### Diagnostic complexity and broader implications

This case exemplifies the diagnostic complexity of APSGN with overlapping features of systemic autoimmune disease. The rare combination of nephrotic-range proteinuria, serosal involvement, severe hypertension, and combined C3/C4 hypocomplementemia mimicked SLE and MPGN. Although SARS-CoV-2 PCR was positive at presentation, the markedly elevated antistreptolysin O titers, classical nephritic presentation, and expected complement recovery strongly support post-streptococcal glomerulonephritis as the primary etiology. SARS-CoV-2 infection was noted at presentation but is best interpreted as a coincidental finding rather than a primary etiologic factor. However, integration of clinical context, and serologic data, enabled accurate diagnosis and avoided unnecessary immunosuppression.

## Conclusion

This case highlights an uncommon presentation of PSGN in which severe extrarenal findings and atypical laboratory abnormalities mimicked systemic autoimmune disease and influenced diagnostic decision-making. While PSGN typically presents as a self-limited renal condition, this patient exhibited dual serositis—simultaneous pleural and pericardial effusions—an exceedingly rare feature more commonly associated with systemic autoimmune diseases such as systemic lupus erythematosus. The coexistence of nephrotic-range proteinuria, severe hypertension, and combined hypocomplementemia (low C3 and C4) further mimicked autoimmune glomerulonephritides, prompting an extensive diagnostic workup. Renal biopsy was deferred in view of clinical resolution of symptoms including hypertension, proteinuria, hematuria and hypocomplementemia. Renal biopsy is generally reserved for patients with atypical features, including persistent hypocomplementemia beyond 8–12 weeks, progressive renal dysfunction, or failure to improve clinically. In this patient, nephrotic-range proteinuria, dual hypocomplementemia, positive ANA, and serosal involvement (pleural and pericardial effusions) initially raised concern for alternative immune-mediated glomerulopathies, making biopsy a consideration. The differential diagnoses that prompted consideration of renal biopsy included lupus nephritis, membranoproliferative glomerulonephritis, and C3 glomerulopathy, given the presence of nephrotic-range proteinuria, dual hypocomplementemia, positive ANA, and serosal involvement. However, given preserved renal function, rapid clinical improvement, normalization of complement levels, and resolution of proteinuria within weeks, biopsy was deferred.

This case adds to the limited pediatric literature describing prominent serosal involvement (pleural and pericardial effusions) at the initial presentation of post-streptococcal glomerulonephritis and highlights how transient complement abnormalities and extrarenal findings may mimic systemic autoimmune disease, influencing biopsy decisions. It serves as a clinically relevant example of how a common pediatric renal disease can present with uncommon systemic features, creating a challenging diagnostic scenario.

## Data Availability

The original contributions presented in the study are included in the article/Supplementary Material, further inquiries can be directed to the corresponding author.

## References

[1] Ong LT. Management and outcomes of acute post-streptococcal glomerulonephritis in children. World J Nephrol. (2022) 11(5):139–45. 10.5527/wjn.v11.i5.139 36187464 PMC9521512

[2] Skrzypczyk P Ofiara A Wąsik M Pańczyk-Tomaszewska M. Acute post-streptococcal glomerulonephritis—immune-mediated acute kidney injury: case report and literature review. Cent Eur J Immunol. (2021) 46(4):516–23. 10.5114/ceji.2021.112244 35125952 PMC8808306

[3] Bajracharya P Khadgi A Paudel R Singh R Paudel N. Acute post-streptococcal glomerulonephritis in a pediatric population: a five-year retrospective study. Cureus. (2024) 16(3):e56082. 10.7759/cureus.56082 38618409 PMC11009898

[4] Demircioglu Kılıc B Akbalık Kara M Buyukcelik M Balat A. Pediatric post-streptococcal glomerulonephritis: clinical and laboratory data. Pediatr Int. (2018) 60(7):645–50. 10.1111/ped.13587 29729114

[5] Ranawaka R Dayasiri K Sandakelum U Nelson D Gamage M. Post-streptococcal acute glomerulonephritis in children: association between proteinuria levels and renal outcomes. World J Clin Pediatr. (2025) 14(1):100885. 10.5409/wjcp.v14.i1.100885 40059899 PMC11686579

[6] Loza S Tallman B Hanson K Rainey S. A 15-year-old with chest pain: an unexpected etiology. SAGE Open Med Case Rep. (2022) 10:2050313X211069026. 10.1177/2050313X211069026 35070318 PMC8777365

[7] Gunasekaran K Krishnamurthy S Mahadevan S Kumar AP Narayanan P. Clinical characteristics and outcome of post-infectious glomerulonephritis in children in Southern India: a prospective study. Indian J Pediatr. (2015) 82(10):896–903. 10.1007/s12098-015-1752-0 25893528

[8] Duong MD Reidy KJ. Acute postinfectious glomerulonephritis. Pediatr Clin North Am. (2022) 69(6):1051–78. 10.1016/j.pcl.2022.08.001 36880922

[9] Grech P Mangion J Vella S. Post-streptococcus mitis infection polyserositis. BMJ Case Rep. (2021) 14(1):e236704. 10.1136/bcr-2020-236704 33472801 PMC10577740

[10] Sethi S Fervenza FC Zhang Y Zand L Meyer NC Borsa N Atypical postinfectious glomerulonephritis is associated with abnormalities in the alternative pathway of complement. Kidney Int. (2013) 83(2):293–9. 10.1038/ki.2012.384 23235567 PMC3561505

[11] Dagan R Cleper R Davidovits M Krause I Garty BZ. Post-infectious glomerulonephritis in pediatric patients over two decades: severity-associated features. Isr Med Assoc J. (2016) 18(6):336–40. PMID: 27468526 27468526

[12] Brant Pinheiro SV de Freitas VB de Castro GV Rufino Madeiro BC de Araújo SA Silva Ribeiro TF Acute post-streptococcal glomerulonephritis in children: a comprehensive review. Curr Med Chem. (2022) 29(34):5543–59. 10.2174/0929867329666220613103316 35702785

[13] Wyatt RJ Forristal J West CD Sugimoto S Curd JG. Complement profiles in acute post-streptococcal glomerulonephritis. Pediatr Nephrol. (1988) 2(2):219–23. 10.1007/BF00862594 3153014

[14] Chehade H Rotman S Frémeaux-Bacchi V Aubert V Sadallah S Sifaki L Blockade of C5 in severe acute postinfectious glomerulonephritis associated with anti-factor H autoantibody. Am J Kidney Dis. (2016) 68(6):944–8. 10.1053/j.ajkd.2016.06.026 27683044

